# The Pectin Lyases in *Arabidopsis thaliana*: Evolution, Selection and Expression Profiles

**DOI:** 10.1371/journal.pone.0046944

**Published:** 2012-10-09

**Authors:** Jun Cao

**Affiliations:** Institute of Life Science, Jiangsu University, Zhenjiang, Jiangsu, P.R. China; Virginia Tech, United States of America

## Abstract

Pectin lyases are a group of enzymes that are thought to contribute to many biological processes, such as the degradation of pectin. However, until this study, no comprehensive study incorporating phylogeny, chromosomal location, gene duplication, gene organization, functional divergence, adaptive evolution, expression profiling and functional networks has been reported for *Arabidopsis*. Sixty-seven pectin lyase genes have been identified, and most of them possess signal sequences targeting the secretory pathway. Phylogenetic analyses identified five gene groups with considerable conservation among groups. Pectin lyase genes were non-randomly distributed across chromosomes and clustering was evident. Functional divergence and adaptive evolution analyses suggested that purifying selection was the main force driving pectin lyase evolution, although some critical sites responsible for functional divergence might be the consequence of positive selection. A stigma- and receptacle-specific expression promoter was identified, and it had increased expression in response to wounding. Two hundred and eighty-eight interactions were identified by functional network analyses, and most of these were involved in cellular metabolism, cellular transport and localization, and stimulus responses. This investigation contributes to an improved understanding of the complexity of the *Arabidopsis* pectin lyase gene family.

## Introduction

Pectins are major primary cell walls components of land plants that are important for maintaining cellular structural integrity [1], [2]. Pectins are a family of complex polysaccharides with 1,4-linked α-D-galactosyluronic acid residues [1], and they can be degraded by pectinases. Pectinases are classified by substrate specificity or mode of action into various classes, such as pectin esterase (EC 3.1.1.11), polygalacturonase (EC 3.2.1.15), pectate lyase (EC 4.2.3.2) and pectin lyase (EC 1.4.2.10). Pectin esterases remove methoxyl and acetyl residues of polygalacturonic acids. Polygalacturonases degrade polygalacturonan by hydrolysis of the glycosidic bonds that link galacturonic acid residues. Pectate lyases are responsible for the eliminative cleavage of pectate, thereby yielding oligosaccharides with 4-deoxy-α-D-mann-4-enuronosyl groups at their non-reducing ends. Pectin lyases are the only known pectinase capable of degrading pectin polymers directly via a β-elimination mechanism that results in the formation of 4,5-unsaturated oligogalacturonides without methanol production [3]. This is very important because methanol’s toxicity and unpleasant, volatile off flavors are a concern for the paper, food and textile industries [4], [5]. Correlative studies about production, biochemical characterization, and applications of pectin lyases were recently reviewed [6].

Structurally, pectin lyase include an asparagines ladder and amino acid stacks and can fold into a parallel β-sheet similar to those in pectate lyase, despite about 17% sequence identity between them [3], [7]. In addition, the substrate-binding clefts of these two pectinases are also dominated by aromatic residues and are enveloped by negative electrostatic potential [3], [7]. While the major difference between them is in the conformation of the loop formed by residues 182–187, which might due to the different pH values of crystallization [3], [7].

Pectinases have multiple biological functions. Pectate lyases can act as extracellular virulence agents [8], [9], and their role in the release of cell wall oligogalacturonides is important for activation of plant defense mechanisms [10]. Furthermore, pectate lyases may be important for fruit ripening and softening [11], as well as plant growth and development [9]. Similarly, polygalacturonases contribute to pectin disassembly during many stages of plant development, such as those that require cell separation [12]. Polygalacturonase genes also have roles in pollen maturation and pollen tube growth, as well as intine and exine formation [13], [14], [15]. In addition, like pectate lyase and polygalacturonase, pectin lyase can enhance the reconstituted expansin-induced extension of the apical (elongating) segments of cucumber hypocotyls [16].

Most pectin lyases are produced by microorganisms (such as *Aspergillus*, *Penicillium* and *Fusarium*). In these microorganisms, expression of pectin lyase gene is generally induced by medium pH, carbon sources and pectin, and is generally repressed by glucose [17], [18]. Limited information is available on the evolution and expression of pectin lyase genes in plants. Because phylogenetic analyses can be the basis for molecular and biochemical analyses of protein families, I performed genome-wide research on the pectin lyase gene family in *Arabidopsis*. Analyses of sequence phylogeny, gene organization, functional divergence, adaptive evolution, expression profiling, functional networks were performed to provide insights into the evolutionary mechanisms of *Arabidopsis*’ pectin lyase protein family.

## Results and Discussion

### Identification and Characterization of the Pectin Lyase Gene Family in *Arabidopsis*

To identify members of the pectin lyase gene family in *Arabidopsis*, I first searched relevant databases using the corresponding *Arabidopsis* protein sequence (AT1G17150) as query. Additional searches were also performed based on keyword querying. All protein sequences with expect value ≤1e−05 related to this family were retrieved from TAIR and NCBI. Other more divergent proteins (such as, QRT3, AUX1, PHO2 etc.) with expect value >1e−05 were not included in this analysis. The *Arabidopsis* sequences returned from such searches were confirmed as encoding pectin lyases using the CDD (Conserved Domain Database) [19], [20] and Pfam (http://pfam.sanger.ac.uk/) databases. Except for one gene (*AT2G33160*), all pectin lyases contained only the polygalacturonase domain. AT2G33160 contained not only the common polygalacturonase domain, but also the RNase H domain. Acquisition of this domain is likely to reflect a functional increase. As a result, 67 pectin lyase genes were identified in *Arabidopsis* (Table 1). The pectin lyase genes in *Arabidopsis* encoded for polypeptides ranging from 332 to 664 amino acids in length, with predicted pIs ranging from 4.78 to 9.84. Further analyses using protein subcellular localization prediction software TargetP [21] and PredoTar (http://urgi.versailles.inra.fr/predotar/predotar.html) predicted the probable protein localization for each of the different candidate pectin lyases in *Arabidopsis*. It was found that over 85% *Arabidopsis* pectin lyase proteins possess signal sequences for targeting the secretory pathway. Five pectin lyases (AT3G06770, AT4G32375, AT4G32380, AT5G27530 and AT5G44830) did not contain any known protein targeting motif. Another five members (AT1G02460, AT1G10640, AT1G19170, AT1G48100 and AT1G60590) were predicted to be targeted to mitochondria (Table 1). Proteins inhibited in endoplasmic reticulum (ER) are usually experience some processing steps, such as protein folding, glycosylation, disulfide bond formation and rearrangement in ER, and are finally transported to their destinations when the N-terminus signal peptides are removed. To identify the potential signal peptides in pectin lyase precursors, SignalP 4.0 server [22] was used. The results indicated that 50 members of pectin lyases possess signal peptide. The length of about 76% signal peptides in *Arabidopsis* pectin lyases is 20∼24 amino acid residues (Table 1). AT5G14650 has the maximum length signal peptide (about 29 amino acid residues). However, signal peptide of AT3G27790 only has 17 residues in N-ternimal extension (Table 1).

**Table 1:** Targeting and signal peptide predictions of 67 *Arabidopsis* pectin lyases using either TargetP V1.1, PredoTar V1.03 and SignalP 4.0.

				TargetP V1.1 Prediction Results					PredoTar V1.03 Prediction Results					SignalP 4.0 Prediction Results
Name	Length	Mw	pI	cTP	mTP	SP	Other	Loc	Mitochondrial	Plastid	ER	Elsewhere	Prediction	Signal peptide position
AT1G02460	491	53462.91	6.00	0.081	0.286	0.063	0.059	M	0.11	0.03	0.18	0.71	none	1∼27
AT1G02790	422	44429.83	8.51	0.010	0.131	0.741	0.066	S	0.20	0.01	0.05	0.76	none	–
AT1G05650	394	41571.39	9.39	0.032	0.005	0.977	0.043	S	0.01	0	0.99	0.01	ER	1∼23
AT1G05660	394	41853.86	9.33	0.009	0.012	0.984	0.030	S	0.01	0	0.99	0.01	ER	1∼23
AT1G10640	532	58347.76	8.87	0.003	0.389	0.361	0.008	M	0.38	0	0.97	0.02	ER	–
AT1G17150	402	43890.30	8.59	0.021	0.057	0.937	0.071	S	0.01	0	0.99	0.01	ER	1∼24
AT1G19170	506	56347.40	9.09	0.022	0.888	0.009	0.470	M	0.03	0.09	0	0.88	none	–
AT1G23460	460	49570.77	5.10	0.008	0.030	0.986	0.050	S	0.02	0	0.99	0.01	ER	1∼22
AT1G43080	404	44244.54	9.02	0.217	0.061	0.769	0.007	S	0.09	0	0.82	0.16	ER	1∼22
AT1G43090	444	49068.30	9.10	0.146	0.06	0.826	0.010	S	0.07	0	0.89	0.10	ER	1∼22
AT1G43100	444	49147.34	9.13	0.232	0.064	0.794	0.008	S	0.09	0	0.82	0.16	ER	1∼22
AT1G48100	475	51352.61	4.80	0.021	0.403	0.373	0.017	M	0.03	0	0.99	0.01	ER	–
AT1G56710	434	47442.88	6.68	0.014	0.049	0.971	0.024	S	0.01	0	0.99	0.01	ER	1∼24
AT1G60590	540	59631.01	6.44	0.016	0.224	0.056	0.134	M	0.15	0.04	0.06	0.77	none	–
AT1G65570	397	42717.74	6.11	0.010	0.034	0.968	0.045	S	0.01	0.03	0.99	0.01	ER	1∼20
AT1G70500	468	50900.45	5.42	0.020	0.020	0.980	0.072	S	0.02	0	0.99	0.01	ER	1∼22
AT1G78400	404	44118.83	9.73	0.063	0.012	0.993	0.010	S	0.02	0.12	0.99	0.01	ER	1∼23
AT1G80140	336	36045.04	5.90	0.018	0.108	0.670	0.276	S	0.01	0	0.08	0.90	none	–
AT1G80170	444	48171.67	8.67	0.010	0.072	0.890	0.005	S	0.02	0	0.99	0.01	ER	1∼28
AT2G15450	404	44080.22	8.69	0.038	0.023	0.964	0.009	S	0.01	0.03	0.98	0.02	ER	1∼22
AT2G15460	402	43828.06	8.87	0.028	0.031	0.968	0.006	S	0.02	0	0.99	0.01	ER	1∼22
AT2G15470	404	44102.32	8.77	0.023	0.037	0.962	0.006	S	0.02	0	0.99	0.01	ER	1∼22
AT2G23900	477	51644.59	8.78	0.023	0.021	0.871	0.221	S	0.13	0.08	0.13	0.7	none	–
AT2G26620	402	43981.35	8.87	0.020	0.058	0.932	0.007	S	0.04	0	0.99	0.01	ER	1∼22
AT2G33160	664	74972.24	8.79	0.007	0.100	0.963	0.063	S	0.02	0	0.99	0.01	ER	1∼21
AT2G40310	404	44085.37	8.82	0.061	0.031	0.898	0.011	S	0.03	0	0.99	0.01	ER	1∼22
AT2G41850	433	46622.70	8.98	0.010	0.147	0.878	0.006	S	0.02	0	0.99	0.01	ER	1∼24
AT2G43860	405	43462.33	6.38	0.005	0.019	0.976	0.054	S	0.02	0	0.99	0.01	ER	1∼26
AT2G43870	384	40810.48	8.56	0.211	0.014	0.747	0.034	S	0.02	0.07	0.99	0.01	ER	1∼18
AT2G43880	394	42735.65	9.84	0.144	0.010	0.973	0.014	S	0.01	0.08	0.83	0.16	ER	1∼27
AT2G43890	392	42008.06	9.78	0.008	0.012	0.961	0.039	S	0.01	0.01	0.99	0.01	ER	1∼25
AT3G06770	377	40807.77	6.04	0.073	0.315	0.142	0.574	–	0.01	0.01	0.21	0.78	possibly ER	–
AT3G07820	391	41681.48	7.03	0.045	0.008	0.896	0.051	S	0.01	0.03	0.97	0.03	ER	1∼22
AT3G07830	397	42913.24	8.92	0.093	0.020	0.737	0.031	S	0.01	0.04	0.97	0.02	ER	1∼22
AT3G07840	401	42591.79	8.31	0.017	0.010	0.973	0.056	S	0.01	0.01	0.99	0.01	ER	1∼23
AT3G07850	444	45600.32	8.63	0.012	0.023	0.974	0.049	S	0.02	0	0.99	0.01	ER	1∼25
AT3G07970	439	48572.20	8.94	0.040	0.010	0.957	0.058	S	0.01	0.01	0.99	0.01	ER	1∼21
AT3G14040	445	45697.42	8.63	0.011	0.019	0.984	0.038	S	0.02	0	0.99	0.01	ER	1∼25
AT3G15720	456	49299.81	5.36	0.002	0.034	0.993	0.032	S	0.01	0	0.99	0.01	ER	1∼23
AT3G16850	455	49124.37	5.46	0.005	0.025	0.965	0.186	S	0.01	0	0.99	0.01	ER	1∼20
AT3G26610	470	51083.50	9.36	0.073	0.006	0.925	0.032	S	0.02	0.01	0.99	0.01	ER	1∼22
AT3G42950	484	54072.53	8.64	0.009	0.114	0.699	0.009	S	0.06	0	0.46	0.50	possibly ER	–
AT3G48950	469	51199.87	8.90	0.076	0.182	0.595	0.015	S	0.03	0	0.99	0.01	ER	1∼22
AT3G57510	431	46572.47	8.20	0.002	0.140	0.986	0.008	S	0.02	0	0.99	0.01	ER	1∼23
AT3G57790	490	54098.59	4.79	0.033	0.110	0.709	0.098	S	0.01	0	0.99	0.01	ER	1∼17
AT3G59850	388	41251.00	9.13	0.061	0.018	0.932	0.046	S	0.02	0.01	0.99	0.01	ER	1∼20
AT3G61490	476	51939.07	5.85	0.141	0.087	0.765	0.065	S	0.01	0.01	0.19	0.80	none	1∼23
AT3G62110	471	51996.84	6.03	0.002	0.233	0.946	0.008	S	0.02	0	0.99	0.01	ER	1∼21
AT4G01890	468	50805.46	8.09	0.019	0.025	0.92	0.059	S	0.04	0	0.94	0.06	ER	–
AT4G13760	404	44214.50	8.90	0.072	0.030	0.922	0.008	S	0.02	0	0.99	0.01	ER	1∼22
AT4G18180	414	44993.98	9.29	0.002	0.011	0.933	0.080	S	0.01	0.01	0.99	0.01	ER	1∼23
AT4G23500	495	54690.82	5.53	0.010	0.032	0.948	0.093	S	0.01	0	0.99	0.01	ER	1∼22
AT4G23820	444	48635.18	8.76	0.040	0.025	0.986	0.034	S	0.03	0	0.99	0.01	ER	1∼20
AT4G32370	342	38134.15	6.79	0.001	0.036	0.989	0.216	S	0.04	0	0.06	0.90	none	–
AT4G32375	486	53315.43	8.67	0.150	0.117	0.243	0.492	–	0.01	0	0	0.99	none	–
AT4G32380	354	38376.29	8.23	0.037	0.056	0.043	0.732	–	0.01	0	0.06	0.93	none	–
AT4G33440	475	52200.51	6.80	0.033	0.079	0.570	0.250	S	0.01	0.01	0.29	0.70	possibly ER	–
AT4G35670	394	42122.42	5.38	0.013	0.02	0.960	0.178	S	0.01	0	0.85	0.15	ER	–
AT5G14650	435	46480.75	8.21	0.001	0.519	0.930	0.007	S	0.09	0	0.97	0.03	ER	1∼29
AT5G17200	421	45234.23	8.77	0.012	0.015	0.990	0.090	S	0.01	0	0.99	0.01	ER	1∼21
AT5G27530	458	49649.8	4.78	0.02	0.157	0.157	0.962	–	0.01	0.02	0	0.98	none	–
AT5G39910	373	41949.53	6.18	0.005	0.038	0.992	0.017	S	0.01	0	0.99	0.01	ER	1∼25
AT5G41870	449	48643.96	6.89	0.014	0.016	0.989	0.023	S	0.01	0	0.99	0.01	ER	1∼27
AT5G44830	332	36363.04	6.44	0.103	0.104	0.053	0.667	–	0.01	0.09	0.07	0.83	none	–
AT5G44840	381	41565.47	7.93	0.006	0.010	0.981	0.111	S	0.01	0.01	0.99	0.01	ER	1∼21
AT5G48140	395	42519.95	9.06	0.013	0.365	0.943	0.019	S	0.01	0	0.99	0.01	ER	1∼23
AT5G49215	449	48758.69	5.40	0.027	0.132	0.825	0.052	S	0.01	0.02	0.99	0.01	ER	1∼21

Mw: molecular weight; pI: isoelectric point; cTP: chloroplast transit peptide; mTP: mitochondrial targeting peptide; SP: secretory pathway signal peptide; S: secretory pathway; M: mitochondria; C: chloroplast; ER: endoplasmic reticulum.

### Phylogenetic Analyses of the Pectin Lyase Genes in *Arabidopsis*

**Figure 1:**
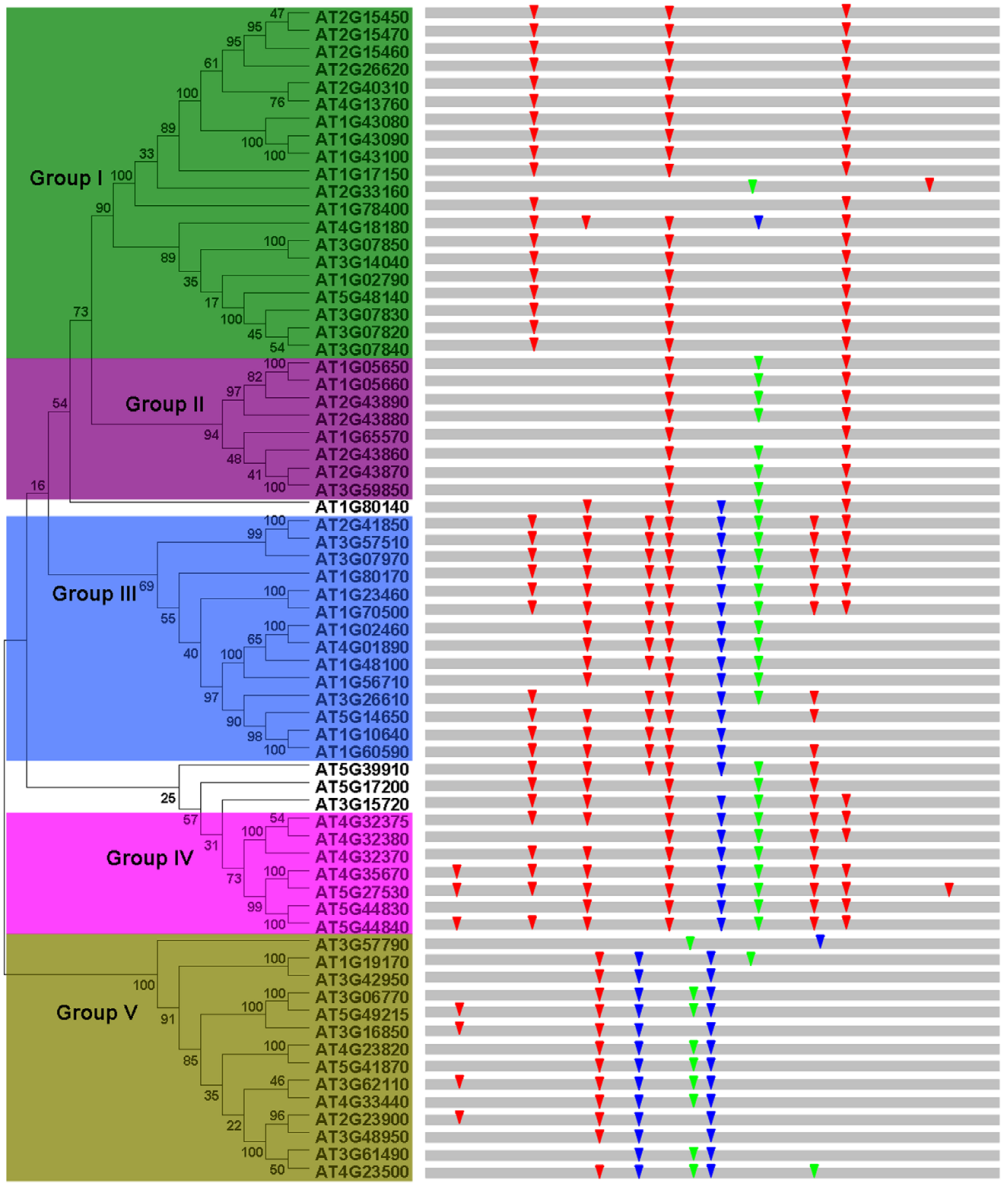
Phylogenetic relationships and exon-intron organization of pectin lyase genes in *Arabidopsis*. Phylogeny tree was constructed using full-length pectin lyase protein sequences. Numbers associated with branches showed bootstrap support values for maximum likelihood analyses. Five major groups designated from I to V were marked with different color. Insertion positions of 0 phase intron, 1 phase intron and 2 phase intron were shown in red, blue and emerald green, respectively.

To examine the phylogenetic relationships in *Arabidopsis* pectin lyase genes, I performed phylogenetic analyses of the pectin lyase protein sequences based on maximum likelihood (ML) method and neighbor joining (NJ) method using MEGA 5 [23]. Tree topology assessed by the ML method was substantially similar to the NJ tree (Figure S1). Based on their phylogenetic relationships, I divided the *Arabidopsis* pectin lyase family into 5 groups, designated from Group I to Group V (Fig. 1). The relationships of *AT1G80140* with other pectin lyase genes, however, could not be confidently determined in my analyses: *AT1G80140* is basal to a large clade consisting of Groups I and II with weak support in the ML analyses, but it forms a clade with Group IV in NJ analyses. Therefore, *AT1G80140* is not classified into any group in this study. In addition, I also excluded *AT3G15720*, *AT5G17200*, and *AT5G39910* out of any groups because of low bootstrap support (Fig. 1). Most of the designated groups were supported by bootstrap values. Moreover, other lines of evidence, such as gene organization (described below), also supported the group classifications established by these analyses. Group I contained 20 members and constituted the largest clade in the pectin lyase phylogeny. Evolutionary relationships between different groups of pectin lyase proteins could not be inferred. By contrast, within each group, strong amino acid sequence and gene organization conservation were evident, suggesting strong evolutionary relationships among subfamily members [24], [25], [26]. Phylogenetic analyses also showed that several pairs of pectin lyase proteins were putative paralogs (Fig. 1). These paralogous pectin lyase members were closely related and had similar structures (described below), indicating that they evolved from relatively recent gene duplications [27], [28]. Evolutionary dates of the segmental duplication events were estimated using *K_s_* as the proxy for time (Table 2). Six of the twenty pairs (*AT2G15450*/*AT2G15470*, *AT2G40310*/*AT4G13760*, *AT1G4309*0/*AT1G43100*, *AT3G07850*/*AT3G14040*, *AT1G05650*/*AT1G05660* and *AT4G44830*/*AT5G44840*) had low *K_s_* values (from 0.00244 to 0.26995), indicative of duplication events occurring within the last 0.08 to 8.99 million years ago. Duplication events of five pairs (*AT2G43870*/*AT3G59850, AT2G41850*/*AT3G57510, AT1G23460*/*AT1G70500, AT4G32375*/*AT4G32380* and *AT3G06770*/*AT5G49215*) occurred within the last 21.28 to 27.17 million years, near the time of large-scale duplication events (polyploidy or aneuploidy) [29], implying that these pairs might have come from these large-scale duplication events. The earliest observed segmental duplication event occurred in the pectin lyase (*AT3G61490*/*AT4G23500*) of *Arabidopsis* around 63.67 million years ago (Table 2).

**Table 2:** Inference of duplication time in paralogous pairs.

Paralogous pairs	*K_a_* 1	*K_s_* 2	*K_a_/K_s_* 3	Data (million years ago)
*AT2G15450*/*AT2G15470*	0.00775	0.01349	0.57450	0.45
*AT2G40310*/*AT4G13760*	0.04751	0.26995	0.17599	8.99
*AT1G43090*/*AT1G43100*	0.00528	0.00244	2.16393	0.08
*AT3G07850*/*AT3G14040*	0.01079	0.07183	0.15052	2.40
*AT3G07820*/*AT3G07840*	0.10929	0.44046	0.24813	14.68
*AT1G05650*/*AT1G05660*	0.05312	0.22517	0.23591	7.51
*AT2G43870*/*AT3G59850*	0.10671	0.81524	0.13089	27.17
*AT2G41850*/*AT3G57510*	0.20058	0.64670	0.31016	21.56
*AT1G23460*/*AT1G70500*	0.14834	0.63845	0.23234	21.28
*AT1G02460*/*AT4G01890*	2.20793	1.07872	2.04680	35.96
*AT1G10640*/*AT1G60590*	0.13571	0.91833	0.14778	30.61
*AT4G32375*/*AT4G32380*	0.32010	0.68921	0.46445	22.97
*AT4G35670*/*AT5G27530*	0.16886	0.45074	0.37463	15.02
*AT4G44830*/*AT5G44840*	0.05001	0.13977	0.35780	4.66
*AT1G19170*/*AT3G42950*	0.18979	1.73433	0.10943	57.81
*AT3G06770*/*AT5G49215*	0.15024	0.70890	0.21193	23.63
*AT4G23820*/*AT5G41870*	0.25535	1.61156	0.15845	53.72
*AT3G62110*/*AT4G33440*	0.44261	1.74450	0.25372	58.15
*AT2G23900*/*AT3G48950*	0.16422	0.98030	0.16752	32.68
*AT3G61490*/*AT4G23500*	0.29295	1.91000	0.15338	63.67

1 *K_a_* is the number of non-synonymous substitutions per non-synonymous site.

2 *K_s_* is the number of synonymous substitutions per synonymous site.

3 *K_a_*/*K_s_* is the ratios of nonsynonymous (*K_a_*) versus synonymous (*K_s_*) mutations. In molecular evolution, it can be used as an indicator of selective pressure acting on a protein-coding gene.

### Exon-intron Organization, Motif Distribution, and Conserved Tertiary Structure for Pectin Lyase Members of *Arabidopsis*

Exon-intron organization has been used as supporting evidence for evolutionary relationships among genes or organisms [30]. To investigate the mechanisms of structural evolution of pectin lyase paralogs in *Arabidopsis*, I compared the exon-intron structures of individual *Arabidopsis* pectin lyase genes. Fig. 1 provided a detailed illustration of the distribution and position of introns within each of the pectin lyase paralogs. From Fig.1, it can be seen that the positions and phases of some introns are conserved in paralogous genes, whereas others are group-specific. Introns are important and necessary subassemblies of eukaryotic genes because their loss or gain is important for generating structural diversity and complexity. This is the basis for the evolution of multiple gene families. Intron’s potential functions include serving as a source of regulatory elements, their role in exon shuffling and alternative splicing, and as a source of signals for exporting mRNA from the nucleus [31], [32]. It has also been suggested that introns can increase intragenic recombination and moderate the evolutionary rate of genes [33]. In addition, growing evidence has shown a functional link between introns and gene expression [34]. Therefore, it is clear that the different expression profiles (described below) might be relevant to different gene organization among these groups. This result was consistent with our previous researches [35], [36], showing that most pectin lyases in the same group have similar coding sequences and similar exon-intron structures, strongly supporting their close evolutionary relationship. There is a need for further examination of the potential functions of different exon-intron structures.

As stated above, the major domains of pectin lyase proteins in *Arabidopsis* were identified using CDD and Pfam. Results showed that all pectin lyase proteins possessed singular characteristics and a structurally conserved polygalacturonase domain that is essential for their pectinase activity. While these tools are suitable for defining the presence or absence of recognizable domains, they are unable to recognize smaller individual motifs and more divergent patterns. Thus, I used the program MEME (http://meme.sdsc.edu) [37] to study the diversification of pectin lyase genes in *Arabidopsis*. Ten distinct motifs were identified in these genes (Figure S2) (Table S1). As indicated above, phylogenetic analyses broadly divided the pectin lyase genes into five groups. Noticeably, most of the closely related members in each of these groups have common motif compositions, suggesting functional similarities among the pectin lyase proteins within the same group (Figure S2). Most members of Groups I, II, III and IV possess 9 motifs, while most members of Group V have 7 motifs. Six of the motifs (motif 1, motif 2, motif 4, motif 5, motif 6 and motif 8) are shared by all pectin lyase proteins. Whether the motifs that are specific to the Groups I, II, III and IV (motif 3, motif 7 and motif 9) or to Group V (motif 10) have the unique functional roles of pectin lyases remains to be further investigated. As an example, multiple alignments clearly showed highly structured motif 8-6-1 clusters of approximately 120 amino acids in Group I located in the proteins middle section. This was one of the most conserved sections of the *Arabidopsis* pectin lyases (Figure S3). Regardless, conserved motifs in pectin lyase proteins of the same group may provide additional support for the results of phylogenetic analyses. On the other hand, the divergence in motif composition among different groups may indicate that they are functionally diversified.

I also estimated the tertiary structures of different pectin lyase groups. The results indicated that all *Arabidopsis* pectin lyases adopt very similar tertiary structures (Figure S2). In addition, I examined the relationships between sequence diversity, motif construction and protein tertiary structure, and found that while there was an increase in sequence diversity or motif construction (as stated above), and pectin lyases in Group V possess different motif constructions from members of other groups. Over time, this variation has a tendency to be limited for protein tertiary structures. If one motif in part of a structure undergoes sequence change, sequence change may also occur in other motif(s) so as to avoid disruption of the protein structure. It has been suggested that correlated sequence or motif evolutions could be a means of maintaining optimal structural and functional integrity [38]. Therefore, I suggest that compensatory changes or co-evolutionary mechanisms also exist in *Arabidopsis* pectin lyase genes.

### *Arabidopsis* Pectin Lyase Gene Chromosomal Location and Duplication Events

**Figure 2:**
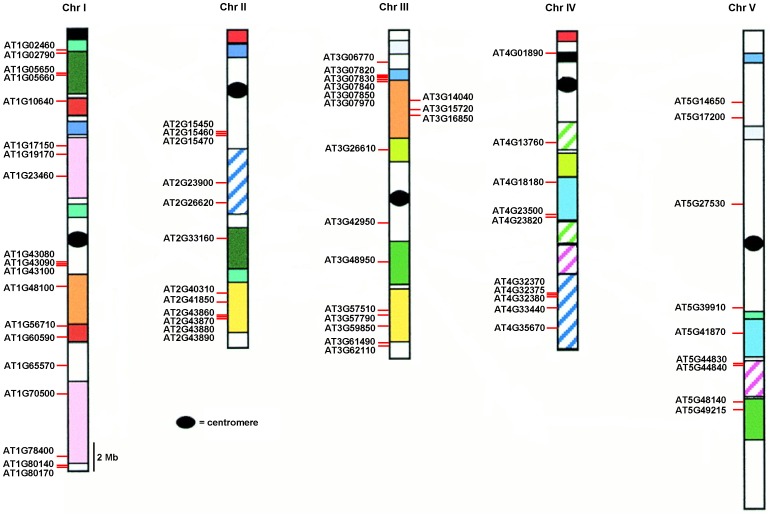
Chromosomal locations of the pectin lyase genes. 67 pectin lyase genes mapped to the five *Arabidopsis* chromosomes are shown. Paralogous regions in the putative ancestral constituents of the *Arabidopsis* genome are depicted using the same colors according to Blanc et al. (2000) [41].

Chromosomal macro- and micro-scale duplication and rearrangement are thought to be the major modes of genome evolution [29], [39], [40]. To investigate the relationship between pectin lyase genes and potential gene duplication within the genome, I compared the locations of pectin lyase genes in duplicated chromosomal blocks that were previously identified in *Arabidopsis* [41]. Pectin lyase genes are unevenly distributed among *Arabidopsis* 5 chromosomes, relative to corresponding duplicate genomic blocks (Fig. 2). Within identified duplication events, only two pairs (*AT2G41850*/*AT3G57510* and *AT4G23820*/*AT5G41870*) are retained as duplicates, whereas all others lack corresponding duplicates. This suggests that dynamic changes occur following segmental duplication that causes the loss of many genes. Therefore, segmental duplication is not the major factor that contributed to expansion of the pectin lyase gene family in *Arabidopsis*. Interestingly, I found that 21 of 67 pectin lyase members are located in tandem clusters on the chromosomes. The two largest pectin lyase gene clusters are located on chromosomes II and III, and each contains four tandem arrayed members, i.e. *AT2G43860*, *AT2G43870*, *AT2G43880* and *AT2G43890* on chromosome II and *AT3G07820*, *AT3G07830*, *AT3G07840* and *AT3G07850* on chromosome III (Fig. 2). Most of these genes form a single phylogenetic clade, suggesting that they may be the result of recent tandem duplications [42], [43]. However, I also found that these clades contained genes from different locations, implying that they may be the consequence of ancient duplication, rearrangement, or retroposition events.

### Functional Divergence Analysis

Could amino acid substitutions have caused adaptive functional diversification? To answer this question, Type-I functional divergence between gene clusters of the *Arabidopsis* pectin lyase family was estimated by posterior analysis using the program DIVERGE [44], [45], which evaluates shifts in rates of evolution and altered amino acid properties. Comparisons of ten pairs of paralogous members’ proteins were carried out and the rate of amino acid evolution at each sequence position was estimated. My results indicated that the coefficient of all functional divergence (θ) values between these groups or classes is less than 1 (Table 3). This indicates significant site-specific selective constraints for most members of the *Arabidopsis* pectin lyase family. This led to group-specific functional evolution after diversification [46], [47]. Moreover, critical amino acid residues responsible of functional divergence were predicted based on site-specific profiles, in combination with suitable cut-off values derived from the posterior probability of each comparison. The results showed distinct differences in the number and distribution of predicted sites for functional divergence within each pair. For example, when the cut-off value is 0.5, no critical amino acid sites are predicted for sequences in Group pairs I/II, I/III,I/IV, II/III, II/IV and III/IV, while approximately 7, 8, 19 and 20 critical amino acids sites are predicted for Group pairs I/V, IV/V, II/V and III/V, respectively. In Table 3, I also found that higher theta values (θ) existed in Group III/V (0.7112), indicating a higher evolutionary rate or site-specific selective relaxation between them. Therefore, because of the different evolutionary rates predicted at some amino acid sites, the pectin lyase genes may be significantly divergent from each other in their functions. During long periods of evolution, different evolutionary rates at specific amino acid sites can spur pectin lyase family genes to evolve new functions after divergence. Thus, functional divergence analysis may reflect the existence of long-term selective pressures.

**Table 3:** Functional divergence estimated in *Arabidopsis* pectin lyase paralogs.

Comparison	θ^1^	SE^2^	LRT^3^	N(0.5)^4^	N(0.7)^4^
Group I/Group II	−0.03263	−0.72954	0	0	0
Group I/Group III	0.088	0.043965	4.006356	0	0
Group I/Group IV	0.001	0.022361	0	0	0
Group I/Group V	0.4752	0.085639	30.78979	7	2
Group II/Group III	0.1688	0.053019	10.13628	0	0
Group II/Group IV	0.0656	0.073899	0.788004	0	0
Group II/Group V	0.6856	0.094327	52.82894	19	9
Group III/Group IV	0.0688	0.047977	2.056406	0	0
Group III/Group V	0.7112	0.083011	73.40285	20	13
Group IV/Group V	0.4456	0.082564	29.12764	8	2

1 θ is the coefficient of functional divergence.

2 SE: standard error.

3 LRT is a likelihood ratio test.

4 N(0.5) and N(0.7) means the numbers of divergent residues when the cut-off value is 0.5 and 0.7, respectively.

### Site-specific Selective Pressures Analysis

The *K_a_/K_s_* ratio measures selection pressure on amino acid substitutions. A *K_a_/K_s_* ratio greater than 1 suggests positive selection and a ratio less than 1 suggests purifying selection [48], [49]. Amino acids in a protein sequence are expected to be under different selective pressures and to have different underlying *K_a_/K_s_* ratios. Detection of selective pressure can indicate selective advantages for altered amino acid sequences in the residues. These selective advantages are essential for understanding functional residues and functional protein shifts [50]. Ratios of nonsynonymous (*K_a_*) versus synonymous (*K_s_*) mutations were used to analyze for positive and negative selection of specific amino acid sites within full-length pectin lyase protein sequences among different groups. This was calculated using Datamonkey, an adaptive evolution server [51], [52]. The results showed that the *K_a_/K_s_* ratios of the sequences from the different pectin lyase groups were significantly different (Table 4). However, despite the differences in *K_a_/K_s_* values, all the estimated *K_a_/K_s_* values were substantially lower than 1, suggesting that the pectin lyase sequences within each of the groups are under strong purifying selection pressure and that positive selection may have acted only on a few sites during the evolutionary process. I performed this analysis using SLAC, FEL and REL methods. The SLAC software detected no positively selected codon sites within the five groups. The REL analyses also found no positively selected sites in Groups III and V, but detected one, two and three positively selected sites in Groups I, II, and IV, respectively. In contrast, FEL analyses identified multiple sites where positive selection occurred (Table 4). I also used PARRIS to test for signatures of selection, but did not identify strong evidence (*P*<0.001) of positive selection in the alignment of pectin lyase coding sequences (Table 5). Therefore, a few sites might have undergone positive selection during evolution, and this might have accelerated functional divergence and the formation of multiple subgroups [53].

**Table 4:** Predicted numbers and locations of codons under positive selection within different pectin lyase groups.

		Positive selection sites			Integrative selection analysis
Gene branches	*Ka*/*Ks*	SLAC	FEL	REL	Total number
Group I	0.3255	–	96,107,111,142,**361**,376,379,435	**361**	8
Group II	0.2607	–	9,69,**141**,**324**,388	**141**,**324**	5
Group III	0.3001	–	5,7,546,557	–	4
Group IV	0.4087	–	183,287,314	131,139,182	6
Group V	0.2706	–	44,69,84,549,551	–	5

Bold codon sites indicate codons that were at least identified with two methods.

**Table 5:** Evidence for positive selection in pectin lyase coding sequences.

Gene branches	Null model Log-likehoods	Alternative mode Log-likehoods	LRT	*P* value	Evidence for positive selection
Group 1	−15164.4	−15164.4	0	1	No
Group 2	−8347.69	−8347.67	0.04	0.980199	No
Group 3	−18795.3	−18795.3	0	1	No
Group 4	−7388.13	−7388.13	0	1	No
Group 5	−18940.5	−18940.5	0	1	No

LRT: likelihood ratio test.

### Differential Expression Profiles of *Arabidopsis* Pectin Lyase Genes

**Figure 3:**
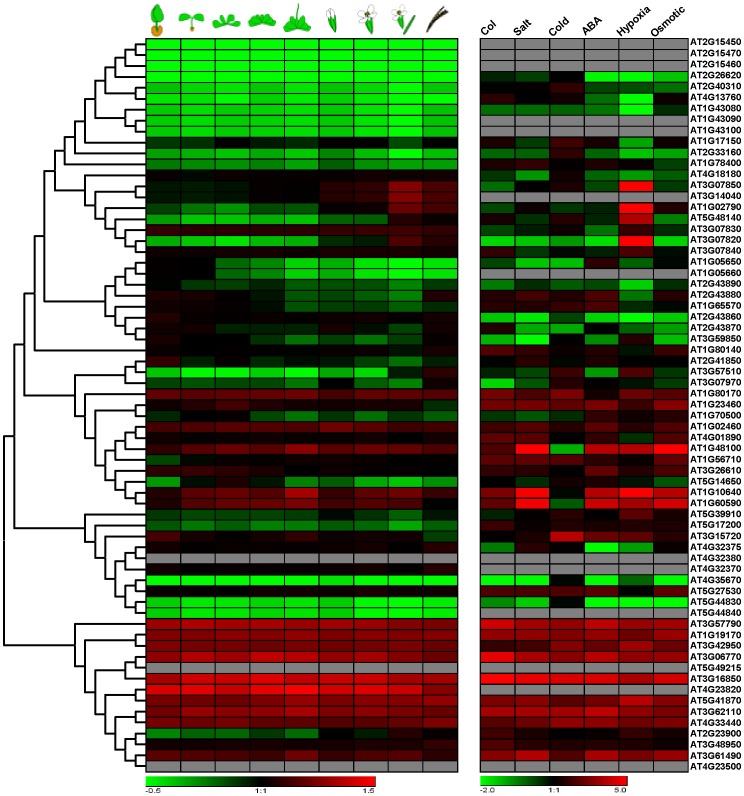
Expression profiles of the *Arabidopsis* pectin lyase genes. (left panel) Dynamic expression profiles of pectin lyase genes for nine different development tissues (germinated seed, seedling, young rosette, developed rosette, bolting, young flower, developed flower, flowers and siliques, and mature siliques). Expression patterns of pectin lyase genes under several abiotic stresses are shown in the right panel.

Expression profiling is a useful tool for understanding gene function. A comprehensive expression analysis was performed using publicly available microarray data for Genevestigator [54], [55] and AtTOME [56]. These analyses indicated that divergent expression profiles are present among pectin lyase members across the nine tissues and developmental stages assessed (Fig. 3). In this study, most genes in Group I were expressed at the lowest levels, genes in Group II, III, and IV at intermediate levels, and genes in Group V at the highest levels. However, some pectin lyases did not to follow this trend. For example, *AT3G07850*, *AT3G14040* and *AT1G07290* in Group I displayed especially high expression levels in flower and silique developmental stages. Nevertheless, *AT2G23900* in Group V displayed low expression levels in stages, from germination to bolting. I also examined the expression patterns of *Arabidopsis* pectin lyases under different stress conditions. Interestingly, several genes such as *AT1G05650*, *AT5G44830* and *AT3G06770* showed low expression levels when treated with salt, whereas a subset of genes, including *AT3G07850*, *AT1G02790*, *AT3G07820* and *AT1G10640*, displayed high expression levels under hypoxic stress (Fig. 3). Similarly, several genes, such as *AT5G48140*, *AT5G14650* and *AT3G62110*, demonstrated depressed expression patterns while under osmotic conditions. As shown in Fig. 3, different expression levels of the pectin lyase genes were found in the nine different growth phases and five abiotic stressors studied, suggesting divergent functions of the pectin lyase members for the developmental process and stress response.

Duplicated genes may have different evolutionary fates, as indicated by divergence in their expression patterns [57]. I also investigated the expression profiles of duplicated pectin lyase gene pairs (identified above) in *Arabidopsis*, and found that most gene pairs did not share similar expression patterns (Fig. 3). This indicates that substantial neofunctionalization may have occurred during the evolution of duplicated genes. During long-term evolution, the expression patterns of the paralogs and duplicated genes have diverged [29]. Such a process may increase the adaptability of duplicated genes to environmental changes, thus conferring a possible evolutionary advantage [58], [59], [60], [61].

**Figure 4:**
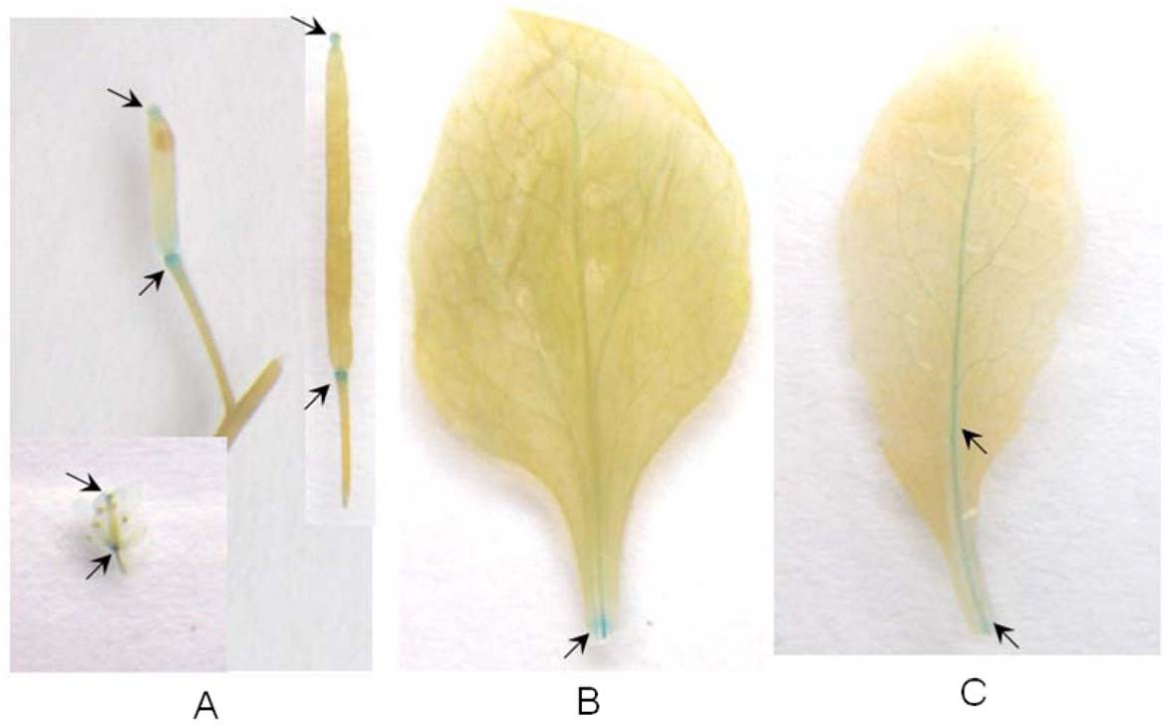
GUS staining of the pBI-P transgenic plants. (A) Strong GUS expression of the *AT1G17150* promoter is observed in floral organs including stigma and receptacle. Moreover, this model of expression can continue during silique development period. (B) Wound can induce the activity of the *AT1G17150* promoter. (C) Wound signal can be transported over time to other areas via vein (12h after wound).

Next, quantitative real-time RT-PCR analysis was conducted to comprehensively examine the differential expression of the *Arabidopsis* Group I pectin lyase genes under wound stress. There were 20 pectin lyase genes for real-time RT-PCR analysis. The results were given in Table 6 and the primer sequences in Table S2. The relative quantification method was used to evaluate the quantitative variation between replicates, and bold indicated up-regulated gene expression under wound treatment. Five pectin lyase genes (*AT2G15470*, *AT2G40310*, *AT4G13760*, *AT1G17150* and *AT3G07830*) in Group I were up-regulated during wound stress (Table 6). The expression of additional members of pectin lyase gene families in Group I was also analyzed, but there transcripts did not change under wound treatment (Table 6). The real-time RT-PCR results indicated that some pectin lyase genes in Group I were up-regulated under wound stress. To clarify the accurately expressional patterns of pectin lyase genes in wound stress responses in plants, I cloned promoter sequence for *AT1G17150*, which has been verified up-regulated gene expression under wound treatment using real-time RT-PCR analysis, and applied a transgenic approach to study its expression in the wound response. As Fig. 4-A shown, strong GUS expression was observed in floral organs, including stigma, and receptacle, (flower stalk or flower abscission zone) using histological staining of GUS activity in promoter-GUS transgenic *Arabidopsis* plants. Moreover, this expression mode continues during the silique developmental period. However, I did not detect observable expression activity in other tissues or organs. Furthermore, strong GUS expression was also observed in regions associated with wounding (Fig. 4-B), and this expression is transported over time to other areas via veins (Fig. 4-C). These results indicated that *AT1G17150* might function during stigma, receptacle, and silique development. Because of increased GUS expression in wounded tissues, I suggested that the *AT1G17150* promoter be responsive to wounding and can activate multiple plant defense systems. In addition, wound signals may be transmitted along veins, thereby increasing the expression or functional scope of this gene.

**Table 6:** Real-time reverse transcriptase-polymerase chain reaction (RT-PCR) for *Arabidopsis* Group I pectin lyase genes under wound stress.

Genes	Fold change (wound/control)
*AT2G15450*	0.955±0.013
*AT2G15470*	**1.298**±0.027
*AT2G15460*	1.012±0.036
*AT2G26620*	1.174±0.019
*AT2G40310*	**1.474**±0.081
*AT4G13760*	**1.287**±0.059
*AT1G43080*	1.036±0.021
*AT1G43090*	1.011±0.014
*AT1G43100*	1.062±0.035
*AT1G17150*	**1.279**±0.021
*AT2G33160*	1.051±0.029
*AT1G78400*	1.133±0.017
*AT4G18180*	1.019±0.062
*AT3G07850*	0.869±0.027
*AT3G14040*	1.076±0.056
*AT1G02790*	1.152±0.071
*AT5G48140*	1.045±0.063
*AT3G07830*	**1.327**±0.084
*AT3G07820*	1.067±0.038
*AT3G07840*	1.051±0.015

Bold indicates up-regulated gene expression under wound treatment.

### Functional Network of the *Arabidopsis* Pectin Lyases

**Figure 5:**
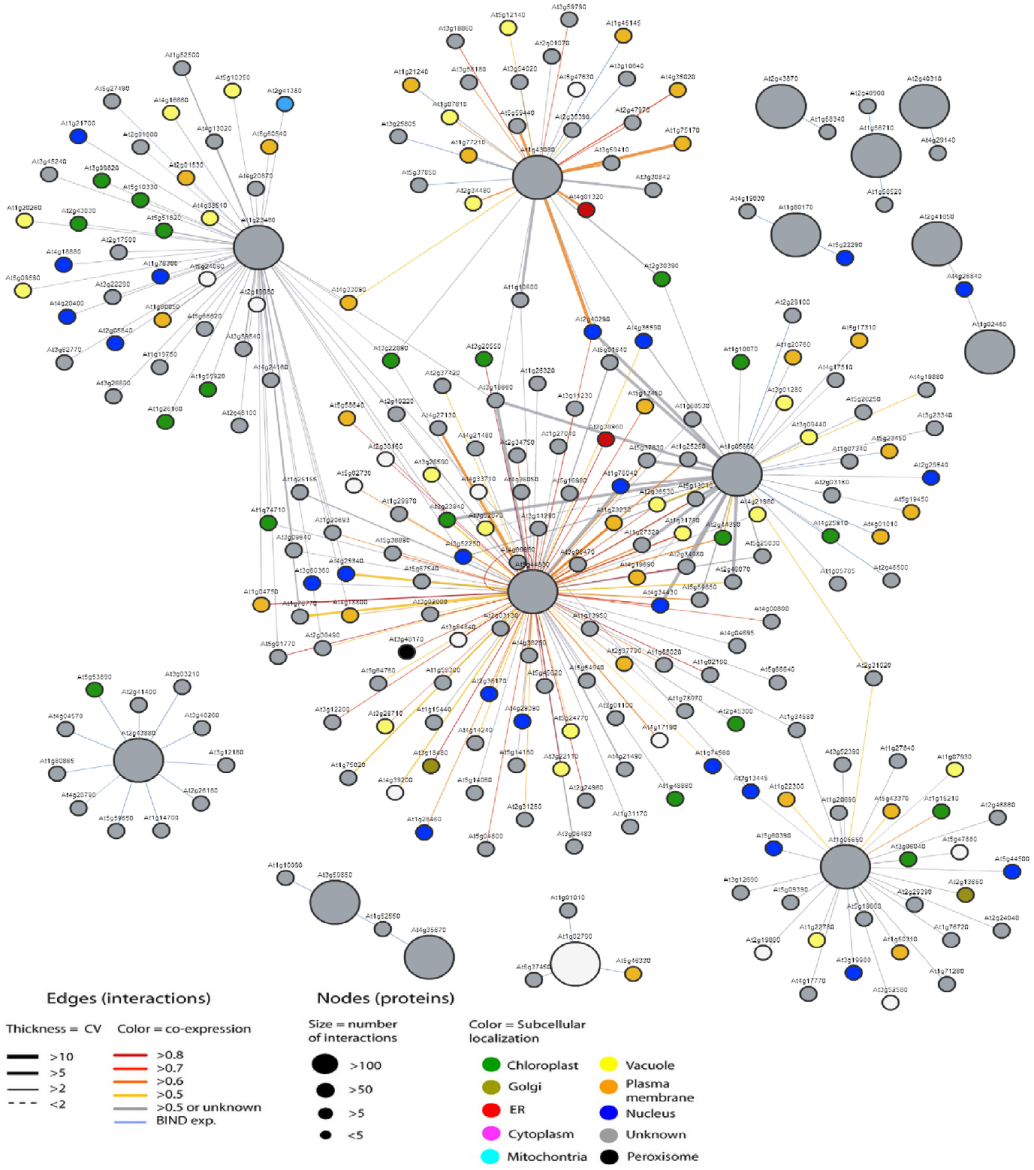
Pectin lyase genes interaction network. Fifteen members of pectin lyase genes are mapped to the protein-protein interaction database. This analysis reveals a total of 235 unique genes (Table S2) that showed 288 interactions, and a network is then assembled based on these interactions.

Genes involved in related biological pathways are usually expressed cooperatively for their functions, and thus information on their interaction is a key to understand the biological systems at the molecular level [62]. To further explore which genes are possibly regulated by members of the pectin lyase family or pathway, a protein-protein interaction network was assembled. The protein-protein interactions were based on experimentally demonstrated or predicted physical interactions [63], and functional interactions suggested by a large number of gene expression analyses [64]. As a result, 15 of 67 pectin lyases were present in the network database, resulting in a total of 235 unique genes that exhibited 288 physical or functional interactions. Therefore, a network for 15 pectin lyase genes was assembled (Fig. 5). Functional categorical analysis of these 235 genes showed that genes involved in cellular metabolism, cellular transport and localization, and stimulus responses were overly represented, compared with the whole genome. Among the 288 interactors identified, 104 and 54 proteins interacted with AT5G44830 and AT1G23460, respectively. Among them, AT5G44830 had 69 unique interactors and AT1G23460 had 36 (Table S3). Some of these interacting genes include *AtSTP9* (*AT1G50310*), *IRT1* (*AT5G43370*), *AT3G09820* (*ADK1*, adenosine kinase 1), *LOS2* (*AT2G36530*), and flagellin-sensitive 2 (*FLS2*, *AT5G46330*). *AtSTP9* encodes a member of sugar transporters that have been linked to pollen germination or pollen tube growth [65]. *IRT1* is identified as a major transporter responsible for high-affinity metal uptake under iron deficiency [66]. *ADK1* is involved in root gravitropism and cap morphogenesis [67], and there is a direct correlation between *ADK1* activity and the level of methylesterified pectin in seed mucilage [68]. *LOS2* encodes a putative enolase and is shown to be a cold-responsive gene [69]. A leucine-rich repeat receptor kinase, *FLS2* acts as a receptor for bacterial pathogen-associated molecular patterns, and contributes to resistance against bacterial pathogens [70]. Multiple transport pathways in plants might be regulated by coordinated expression of different genes. The interaction networks reflect the correlation of the expression pattern of different genes, and are suggestive in tracing the genes in the same pathway. Here, this network analysis has revealed that the function of some pectin lyase proteins might require the participation of various members of sugar transporters (Fig. 5). A pectin lyase, AT1G05650, was found to be interacted with three transporters, AtSTP1 (AT2G13650), AtSTP2 (AT1G07340) and AtSTP9. In addition, two other sugar transporters, AtSTP14 (AT1G77210) and AtSTP12 (AT4G21480) might be the potential interactors of the pectin lyases, AT1G43080 and AT5G44830, respectively. Thus, whether sugar transportation could serve as a link to pectin degradation process was a suggestive direction in further study. The pectin lyase gene was interacted with the same group of transporters, suggesting that they may take part in correlated molecular pathways by interaction with these partners. Plant pathogen resistance is crucial to plant development and reliable production of food. This functional network analysis has revealed that pectin lyase genes may function with pathogen resistance proteins. An important group of enzymes involved in pathogen resistance is leucine-rice repeat proteins kinases (FLS2) [70]. Here, four leucine-rice repeat proteins kinases were identified to be coexpressed with the pectin lyase proteins, suggesting possible interactions between the pectin lyase and FLS genes. Although the exact pathways mediated by these genes were still unclear, one might speculate that these pectin lyase genes play important roles in disease resistance. The characterization of pectin lyase proteins function could therefore open new perspectives for understanding the molecular mechanism of bacterial disease resistance. These findings suggest that differential interaction may assist in the investigation of key regulatory steps in metabolic pathways. The approaches and results reported here would be useful to prioritize candidate genes for further functional genomics studies of *Arabidopsis*.

### Conclusion

This study provided a comparative genomic analysis addressing phylogeny, chromosomal location, gene structure, functional divergence, selective pressures, expression profiling, and functional networks of the pectin lyase gene family in *Arabidopsis*. Phylogenetic analyses revealed five well-supported groups in the pectin lyase family. The exon/intron structure of the pectin lyase genes were highly conserved in each of the groups, indicative of their functional conservation. The pectin lyase genes were non-randomly distributed across the *Arabidopsis* chromosomes, and a high proportion of the pectin lyase genes might be derived from tandem duplications. An additional functional divergence analyses suggested that significant site-specific selective constraints might have acted on most pectin lyase paralogs after gene duplication, leading to subgroup-specific functional evolution. Furthermore, comprehensive analysis of the expression profiles provided insights into possible functional divergence among members of the pectin lyase gene family. A wound-induced promoter for one pectin lyase gene that was expressed in stigma- and receptacle-specific tissues was also confirmed. Functional network analyses identified cellular metabolism, cellular transport and localization, and stimulus response genes. These data may provide valuable information for future functional investigations of this gene family.

## Materials and Methods

### Sequence Retrieval, Identification, and Microarray Analyses

Potential members of the pectin lyase gene family of *Arabidopsis* were identified using multiple database searches. One pectin lyase (*AT1G17150*) gene sequence was retrieved and used as a query in BLASTP searches against the TAIR (The Arabidopsis Information Resource; http://www.arabidopsis.org) using the default settings. PSIBLAST (Position-Specific Iterated BLAST) searches were also performed against the NCBI (National Center for Biotechnology Information) using the same sequence. All protein sequences with expect value ≤1e−05 related to this family were retrieved from TAIR and NCBI and redundancies were removed. TargetP [21] and PredoTar (http://urgi.versailles.inra.fr/predotar/predotar.html)] were used for primary structural analysis of the *Arabidopsis* pectin lyases. SignalP 4.0 server [22] was used to identify the potential signal peptides in pectin lyase precursors. Molecular weights and isoelectric points of pectin lyases were estimated using Compute pI/Mw (http://web.expasy.org/compute_pi/). Genome-wide microarray data were obtained from Genevestigator [54], [55], [71]) and the Arabidopsis Transcriptome Genomic Express Database (http://signal.salk.edu/cgi-bin/atta).

### Phylogenetic Analyses of the *Arabidopsis* Pectin Lyase Gene Family

Multiple sequence alignments of the full-length protein sequences were performed using MUSCLE 3.52 [72], followed by manual comparisons and refinement. Phylogenetic analyses of the pectin lyase proteins based on amino acid sequences were carried out using ML and NJ methods in MEGA v5 [23]. NJ analyses were done using *p*-distance methods, pairwise deletion of gaps, and default assumptions that the substitution patterns among lineages and substitution rates among sites are homogeneous. Support for each node was tested with 1,000 bootstrap replicates. ML analyses were done using a Poisson substitution model, uniform rates among sites, and partial deletion of gaps (95%). Support for each node was also tested with 1,000 bootstrap replicates.

### Chromosomal Location and Gene Structure of the Pectin Lyase Genes

The chromosomal locations of the pectin lyase genes were determined using the Chromosome Map Tool (http://www.arabidopsis.org/jsp/ChromosomeMap/tool.jsp) on TAIR. Gene intron/extron structure information was collected for genome annotations of *Arabidopsis* from TAIR and NCBI (http://www.ncbi.nlm.nih.gov/) databases.

### Protein Tertiary Structure Model-building and Visualization Analysis

The tertiary structure prediction and visualization analysis of pectin lyase proteins were performed with PHYRE2 server (http://www.sbg.bio.ic.ac.uk/~phyre2/html/page.cgi?id=index) [73].

### Inference of Duplication Time

MEGA 5 [23] was used to perform the pairwise alignment of nucleotide sequences of the pectin lyase paralogs with ClustalW (codons). K-Estimator 6.0 program [74] was used to estimate the *K_a_* and *K_s_* values of paralogous genes. Estimates of evolutionary rates are useful for explaining patterns of macroevolution because *K_s_* can be used as a proxy for time when estimating dates of segmental duplication events. The *K_s_* value was calculated for each of gene pair and then used to calculate the approximate date of the duplication event (T = *K_s_*/2λ), assuming clock-like rates (λ) of synonymous substitution (1.5×10^−8^) for *Arabidopsis* [29].

### Functional Divergence Analyses

To estimate levels of functional divergence and predict amino acid residues responsible for functional differences in pectin lyase groups, the coefficients of type-I functional divergence were calculated using the method suggested by Gu [44], [45]. Analyses were carried out using DIVERGE (version 2.0) [44], [45], which uses maximum likelihood procedures to estimate significant changes in site-specific shifts in evolutionary rates or site-specific shifts in amino acid properties after the emergence of two paralogous sequences. The advantage of this method is that it uses amino acid sequences and, therefore, is not sensitive to saturation of synonymous sites. Type-I functional divergence designates amino acid configurations that are highly conserved in gene 1 but highly variable in gene 2, or vice versa, implying that these residues have experienced altered functional constraints [44]. Coefficients of functional divergence significantly greater than 0 indicate site-specific altered selective constraints or radical shifts of amino acid physiochemical properties after gene duplication. Site-specific posterior analysis is also used to predict amino acid residues that may be crucial for functional divergence.

### Site-specific Selection Assessment and Testing

Three methods [SLAC (single likelihood ancestor counting), FEL (fixed-effects likelihood) and REL (random-effects likelihood)] were employed to select individual codons, using the default settings of the Datamonkey web interface [51], [52]. SLAC fits a nucleotide substitution model to the data and then calculates a global *Ka/Ks* ratio. Next, ancestral sequences at each codon are reconstructed using maximum likelihood methods. Finally, expected and observed numbers of synonymous and nonsynonymous substitutions are calculated to infer selection at each codon site. Significance is assessed by using a *P* value derived from a two-tailed binomial distribution. SLAC calculates the expected and observed numbers of synonymous and nonsynonymous substitutions to infer selection. FEL directly estimates *Ka* and *Ks* based on a codon-substitution model, and a likelihood ratio test assesses significance at a level of 0.1. REL is an extension of the site-by-site positive selection analyses implemented in PAML [75]. Notably, it allows synonymous and nonsynonymous substitution rates to vary among codon sites, and uses Bayes factors >50 to determine codon sites (default conditions) [51], [52]. Finally, “integrative selection analysis” was applied to identify the total number of positively selected codons that were detected by at least 1 of the 3 methods [51], [52]. PARRIS [76] was concurrently used to test for signatures of selection, because it allows tree topologies and branch lengths to change across detected recombination breakpoints. In addition, synonymous substitution rates were allowed to vary across codon sites. A null model was fitted to the data, followed by an alternative (selection) model. A likelihood ratio test was then used to compare models and test for evidence for positive selection [76].

### Plant Growth Conditions, Wound Treatments and RNA Isolation

*Arabdiopsis* ecotype Columbia was used in this study. Seeds were sterilized with one rinse in 70% EtOH for 10 min, followed by one rinse in 10% NaClO for 5 min, and then five rinses in sterilized water. Sterilized seeds were sown on Petri dishes containing Murashige-Skoog (MS) salts with 0.75% w/v agar B. Dishes were kept at 4°C for 2 days and then moved to a growth chamber at 22°C and 80% relative humidity under light/night (16h/8h) conditions. Approximately 10 days after germination, seedlings were transplanted to Cornell-mix soil (3∶2:1 peat moss:vermiculite:perlite, v/v/v) and grown in a growth chamber. *Arabidopsis thaliana* ecotype Columbia was soil-grown as described above for 5 weeks. Wounding of soil-grown plants (5 weeks old) was performed by thoroughly crushing with forceps one-half of the rosette leaves. Wounded and nonwounded leaves were harvested at the indicated times and frozen in liquid nitrogen for further total RNA isolation. Total RNA was extracted from by using the Trizol total RNA extraction kit (Sangon, shanghai, China, SK1321) and was treated with RNase free DNase-I to remove genomic DNA.

### Real-time Quantitative RT-PCR

Reverse transcription was performed with total RNA (2 µg) using M-MLV (Sangon, shanghai, China). The cDNA samples were diluted to 8 ng/µL. Triplicate quantitative assays were performed on 1 µL of each cDNA dilution using SYBR Green Master Mix (Sangon, shanghai, China, BS643) with an ABI HT7900 sequence detection system, according to the manufacturer’s protocol. The gene-specific primers were synthesized in Sangon. The amplification of *Atactin-11* (*AT1G12110*) RNA was used as an internal control to normalize all data (forward primer, 5′-CCACATGCTATTCTGCGTTTGGACC-3′; reverse primer, 5′-CATCCCTTACGATTTCACGCTCTGC-3′). The gene-specific primers are given in Table S2. The relative expression levels were calculated using the ddCt method and *Atactin-11* as endogenous control to normalize all data. Fold changes were calculated from the expression data obtained by qRT-PCR in all samples. Quantitative variation was evaluated between replicates.

### Plasmid Construction and Plant Transformation

For constructing the fusing vector of promoter of *AT1G17150*::GUS, the promoter region of *AT1G17150* was amplified using primers At1-1 (5′-AAGCTTGTATACAGGTTTTAGTAGTTGAT-3′, the underlined section is an engineered *Hin*dIII) and AT1-2 (5′-GGATCCAATGCACGGCGCCATCTTCTTCT-3′, the underlined section is an engineered *Bam*HI) on a template of gDNA derived from leaves. The PCR products were cloned into pMD18-T (TaKaRa, Dalian, China) to form pMD-P. The promoter sequence was then released from pMD-P with *Bam*HI and *Hin*dIII, and was subcloned into *Bam*HI and *Hin*dIII sites of pBI121. This formed pBI-P for producing recombinant promoter::GUS fusion vector. The binary vector (pBI-P) was transferred into *Agrobacterium tumefaciens* strain EHA105 and the transformed Agrobacterium cells were used to transform *A. thaliana* ecotype Columbia via the floral dip method [77].

### Histochemical GUS Staining

The histochemical GUS assay was performed in a staining solution containing 0.5 mg/ml 5-bromo-4-chloro-3-indolyl glucuronide in 0.1 M Na_2_HPO_4_, pH 7.0, 10 mM Na_2_EDTA, 0.5 mM potassium ferricyanide/ferrocyanide, and 0.06% Triton X-100 [78]. Samples were infiltrated with staining solution and incubated at 37°C overnight. Staining buffer was removed, and samples were subsequently cleared in 70% EtOH. All observations were recorded with a Sony digital camera.

### Network Assembly

A protein-protein network was assembled using previously described methods [79], [80]. Briefly, the network database includes known protein interaction databases, data from yeast two-hybrid experiments, predicted interactions via orthology and co-citations [63], other literature sources, and a recently reported interaction database derived from analysis of large-scale DNA microarray analyses [64]. Pectin lyase genes were mapped to their corresponding proteins in the network database. Fifty-two pectin lyases were not present in the assembled network database. Resulting interactions were used to build the 15 members interaction network.

## Supporting Information

Figure S1 **Phylogenetic relationships of pectin lyase genes in** ***Arabidopsis*** **.** The molecular phylogeny is constructed using full length pectin lyase protein sequences based on neighbor joining method (left panel) and maximum likelihood method (right panel) using MEGA 5. Numbers associated with branches show bootstrap support values.
(TIF)

Figure S2 **Motif composition and conserved tertiary structures of** ***Arabidopsis*** **pectin lyases.** A schematic representation of conserved motifs (obtained using MEME) in pectin lyase proteins is displayed in the middle panel. Different motifs are represented by different colored boxes. Details of the individual motifs are in the Table S1. Representative tertiary structures of different groups are also shown on the right.
(TIF)

Figure S3 **Multiple sequence alignment of motif 8-6-1 clusters in** ***Arabidopsis*** **Group I pectin lyase members.** Multiple alignment results clearly show the high conserved motif 8-6-1 clusters in *Arabidopsis* Group I pectin lyase members. Secondary structure elements are shown above the alignment.
(TIF)

Table S1 **Motif sequences identified by MEME tools.** Numbers correspond to the motifs were described in Figure S2.
(DOC)

Table S2 **Primers used for real-time quantitative RT-PCR.**
(DOC)

Table S3 **Pectin lyase-interacting proteins identified by using the BAR** ***Arabidopsis*** **Interactions Viewer and their functional categories.**
(XLS)
