# Supplementary figures and images for: The Pectin Lyases in Arabidopsis thaliana: Evolution, Selection and Expression Profiles
Source: PLoS One. 2012 Oct 9;7(10):e46944. doi: 10.1371/journal.pone.0046944 (PMC3467278; doi:10.1371/journal.pone.0046944)

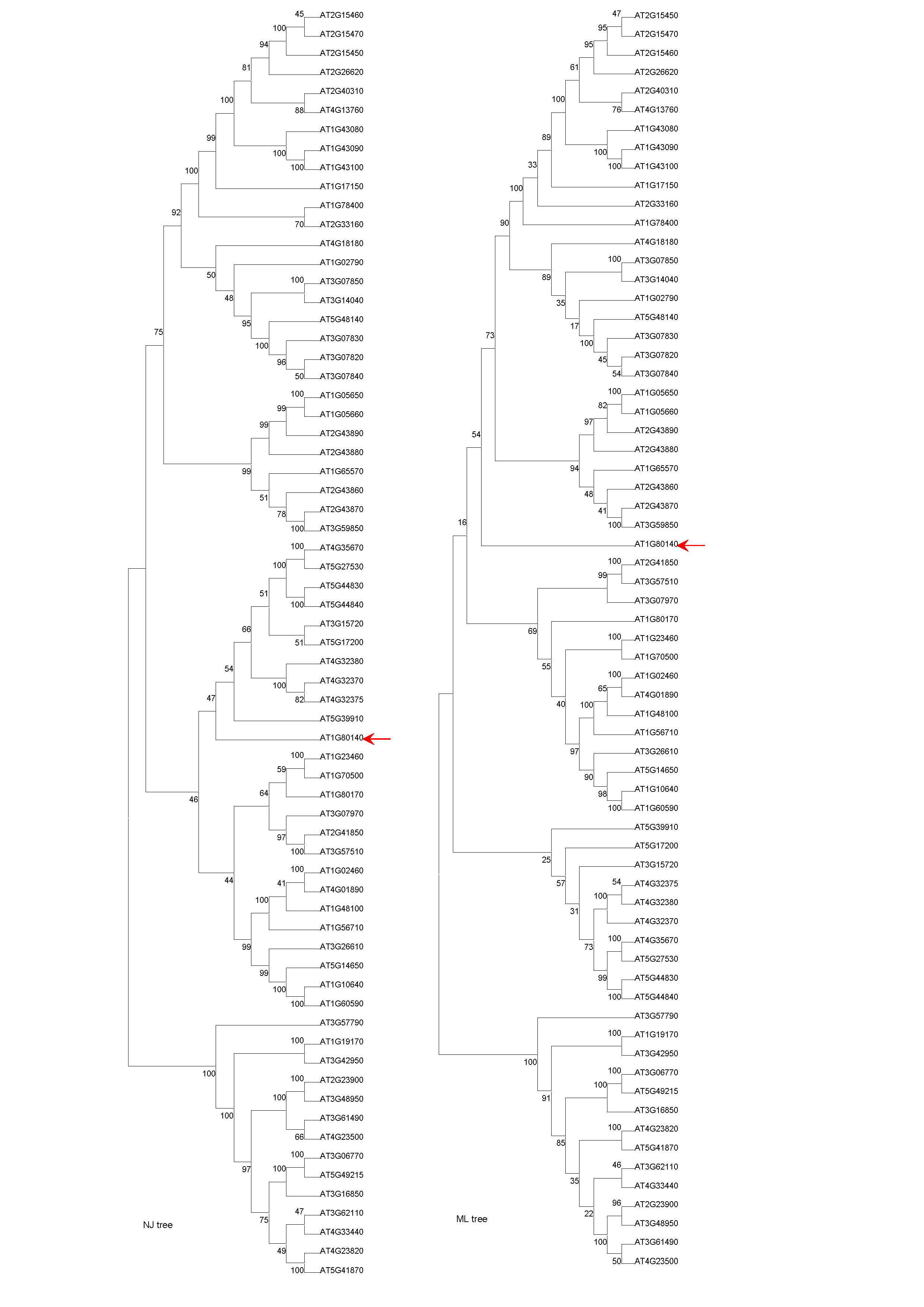

Supplement: Figure S1 — Phylogenetic relationships of pectin lyase genes in Arabidopsis . The molecular phylogeny is constructed using full length pectin lyase protein sequences based on neighbor joining method (left panel) and maximum likelihood method (right panel) using MEGA 5. Numbers associated with branches show bootstrap support values. (TIF) [file pone.0046944.s001.tif]

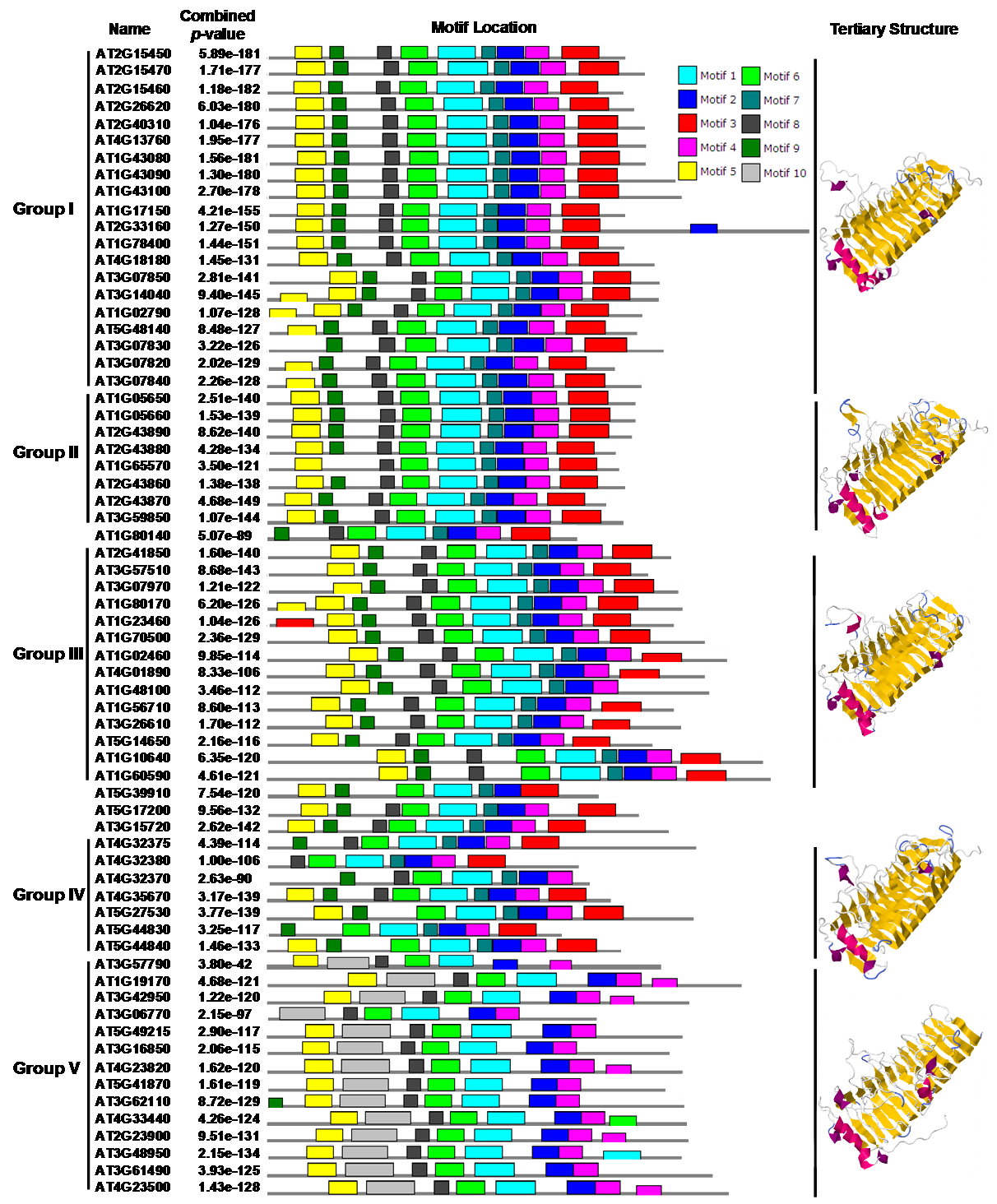

Supplement: Figure S2 — Motif composition and conserved tertiary structures of Arabidopsis pectin lyases. A schematic representation of conserved motifs (obtained using MEME) in pectin lyase proteins is displayed in the middle panel. Different motifs are represented by different colored boxes. Details of the individual motifs are in the Table S1. Representative tertiary structures of different groups are also shown on the right. (TIF) [file pone.0046944.s002.tif]

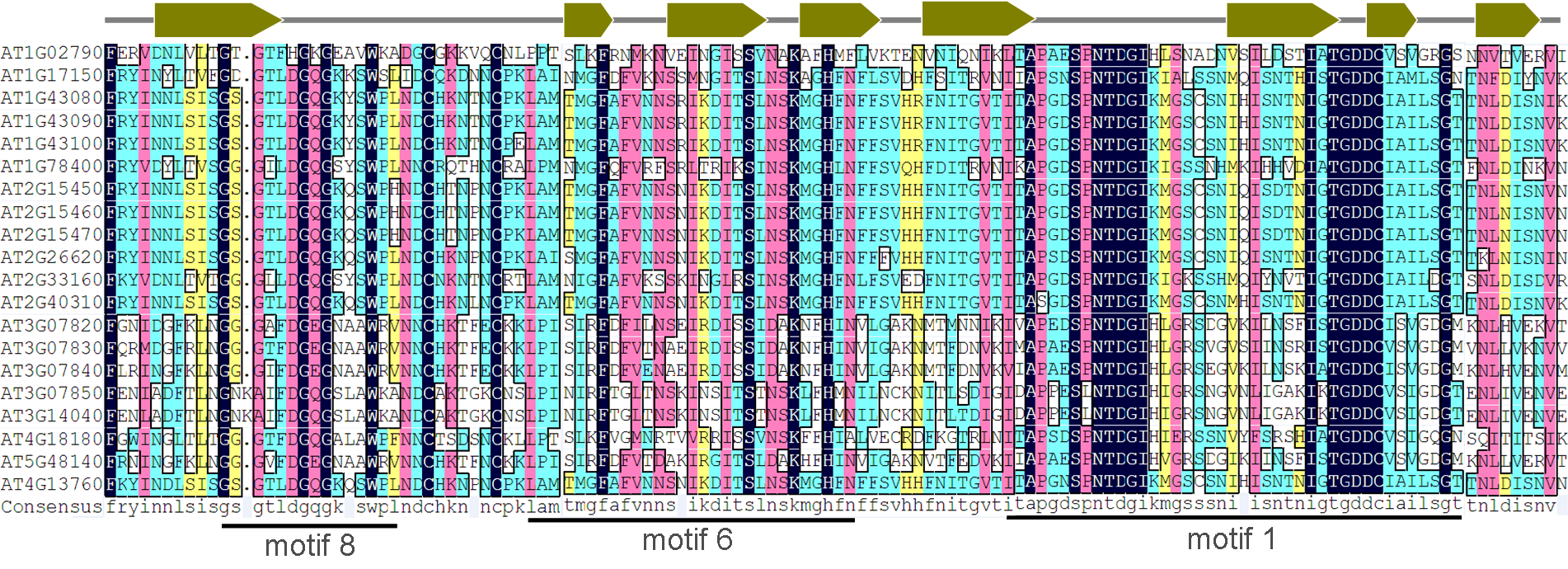

Supplement: Figure S3 — Multiple sequence alignment of motif 8-6-1 clusters in Arabidopsis Group I pectin lyase members. Multiple alignment results clearly show the high conserved motif 8-6-1 clusters in Arabidopsis Group I pectin lyase members. Secondary structure elements are shown above the alignment. (TIF) [file pone.0046944.s003.tif]
