# Supplementary material for: The Pectin Lyases in Arabidopsis thaliana: Evolution, Selection and Expression Profiles
Source: PLoS One. 2012 Oct 9;7(10):e46944. doi: 10.1371/journal.pone.0046944 (PMC3467278; doi:10.1371/journal.pone.0046944)
Supplement: Table S1 — Motif sequences identified by MEME tools. Numbers correspond to the motifs were described in Figure S2. (DOC) [file pone.0046944.s004.doc]

Table S1: Motif sequences identified by MEME tools. Numbers correspond to the motifs were described in Figure S2.

| **Motif** | **E-value** | **Multilevel consensus sequence** |
| --- | --- | --- |
| 1 | 6.7e-1372 | ITAPGDSPNTDGIHIGS[SC][ST]N[VI]xIS[DN][ST]TI[GS]TGDDC[IV][AS]I[GK]SG[TS] |
| 2 | 5.9e-775 | [ES][AG][GD]V[SK][ND][VI]TVR[DN]CT[FI]N[GN][TS]DNG[VI]RIKTW[QP][GS][GSR] |
| 3 | 1.0e-765 | [VI][KQ]I[SQ][ND][VI][KT][FYL]KNI[RY]GTS[AK][NT]KVA[VI][KN]L[QD]CSKS[FV]PC[KT][NGD][IV][EV]LI[DN][IV][NK][LI] |
| 4 | 1.2e-692 | GY[VA][SKR]NIL[FY]ENI[QT][ML]I[ND]V[KG]NPIIIDQxY |
| 5 | 4.5e-728 | VF[ND]VT[SD][FY]GA[KV][GP]DGKTD[ND]TKAFTSAWKAAC |
| 6 | 6.5e-608 | [RL][PA]TA[ILM][GR]FxF[SV]NN[VSI]x[IV][SK][GN][IL]TS[LI][ND]S[PKQ][MQ]FHI[NH] |
| 7 | 5.8e-530 | CGPGHGIS[IV]GSLG[KR][DY] |
| 8 | 1.7e-342 | G[GNS]G[TV][IL]DG[QR]G[KS]xWWPL |
| 9 | 1.1e-230 | P[KG]GT[FY][LY][LV]G[PS][VI][ET]FVGP |
| 10 | 6.0e-346 | VP[PA]G[KR]WLTGSFNLTSH[FLM]TLFL[EA][KR][GD]A[VT]IL[AG][SV]QD[EP]S[EH][WY]P[VL][IV]DPLPSYGRG[RI][ED] |
