# Supplementary material for: The Pectin Lyases in Arabidopsis thaliana: Evolution, Selection and Expression Profiles
Source: PLoS One. 2012 Oct 9;7(10):e46944. doi: 10.1371/journal.pone.0046944 (PMC3467278; doi:10.1371/journal.pone.0046944)
Supplement: Table S2 — Primers used for real-time quantitative RT-PCR. (DOC) [file pone.0046944.s005.doc]

Table S2: Primers used for real-time quantitative RT-PCR.

| **Primers** | **Sequences (5'-3')** |
| --- | --- |
| AT1G02790-F | cggtctatgatattacta |
| AT1G02790-R | gctggtcgtagcgatgatt |
| AT1G17150-F | ctccgaacaacgacgaca |
| AT1G17150-R | atcccattcatgcttgagtt |
| AT1G43080-F | gctatgactatgggatttgc |
| AT1G43080-R | ccgtcagtatttgggctat |
| AT1G43090-F | ccctcacccaccttgtga |
| AT1G43090-R | ggaccattgagtcaatctagaca |
| AT1G43100-F | acaccaccgaggtagaat |
| AT1G43100-R | gtccgactatcagaccaaag |
| AT1G78400-F | ccaagacttccaacgctgat |
| AT1G78400-R | ttgccgccaaacccataa |
| AT2G15450-F | ccgacggtatcaagatgggt |
| AT2G15450-R | ggtgggtgaggacaatacttct |
| AT2G15460-F | cggtacaagcgatggtattc |
| AT2G15460-R | catgttcacaaggtgggtg |
| AT2G15470-F | gctcccggcgatagtcc |
| AT2G15470-R | cccatgtccaggaccaca |
| AT2G26620-F | cacgaactctacaccaccta |
| AT2G26620-R | cggacggaaagactgata |
| AT2G33160-F | ttggagcctttaactgga |
| AT2G33160-R | gtgttcttgcgaggatgt |
| AT2G40310-F | ccgacggtatcaagatgg |
| AT2G40310-R | cacaaggaggatgaggaca |
| AT3G07820-F | gatgaggattgggttgct |
| AT3G07820-R | agtgtcgggagtcttgga |
| AT3G07830-F | gcgtctttgttccctgatg |
| AT3G07830-R | gctccaccactgcctcca |
| AT3G07840-F | tctccacgaaatcaaacct |
| AT3G07840-R | caggcacttctgaaagca |
| AT3G07850-F | gaatgttggaggcgatgc |
| AT3G07850-R | tggaccaggacacggaat |
| AT3G14040-F | cactgtaatcgtgctccc |
| AT3G14040-R | cacaatagcaaacgggac |
| AT4G13760-F | cggtatcaagatgggttca |
| AT4G13760-R | gctcatgttcacaaggtgg |
| AT4G18180-F | atctgtctttgttgcgttgt |
| AT4G18180-R | ccgcctgatctatactttga |
| AT5G48140-F | cgaaaacaccacctccgt |
| AT5G48140-R | ttcccgacaaagagcacc |
